# Self-care practice and glycaemic control amongst adults with diabetes at the Jimma University Specialized Hospital in south-west Ethiopia: A cross-sectional study

**DOI:** 10.4102/phcfm.v4i1.311

**Published:** 2012-05-09

**Authors:** Endalew Hailu, Wudineh H. Mariam, Tefera Belachew, Zewdie Birhanu

**Affiliations:** 1 Department of Nursing, Jimma University, Ethiopia; 2 Department of Population and Family Health, Jimma University, Ethiopia; 3 Department of Health Education and Behavioral Sciences, Jimma University, Ethiopia

## Abstract

**Background:**

The main goal in diabetes care is to improve the patient's quality of life, to maintain satisfactory metabolic control and to retain minimal complications caused by diabetes mellitus (DM). Thus, this study has assessed self-care practice and glycaemic control amongst adults with diabetes mellitus.

**Setting:**

A facility-based study amongst the diabetic follow-up clinic at Jimma University Specialized Hospital in Ethiopia.

**Methods:**

A cross-sectional study was conducted from 01 April to 30 April 30 2010. A total of 343 diabetic patients were selected using a systematic sampling method. The data were collected by structured questionnaires and a medical card review; anthropometric measurement was done by trained nurses.

**Results:**

The study showed that 53% of the respondents had diabetes related knowledge. The study also found that 64% of the respondents were physically less active, and 17% of the respondents were walking on foot for less than 30 minutes per a day. Only 18.1% of the respondents were able to control their Fasting Blood Sugar (FBS) to level below 126 mg/dL.

**Conclusion:**

The present study illustrates that the level of knowledge about diabetes and self-care practices amongst diabetic patients were meager. In addition, it showed that respondents’ level of physical activity, their educational status, and the dose of oral hypoglycaemic agents taken by the respondents significantly affected glycaemic control.

## Abstrait

**Pratique des autosoins et contrôle de la glycémie chez des adultes souffrant de diabète à l'hôpital universitaire spécialisé de Jimma dans le Sud-ouest de l’Éthiopie: Une étude transversale**

**Contexte:**

Le principal objectif de la prise en charge du diabète est d'améliorer la qualité de vie du patient, de maintenir un contrôle métabolique satisfaisant et de limiter les complications causées par le diabète sucré. Cette étude a donc évalué la pratique des autosoins et le contrôle glycémique chez des adultes souffrant de diabète sucré.

**Contexte:**

Une étude basée dans une structure de santé spécialisée dans le suivi des personnes souffrant de diabète à l'hôpital universitaire spécialisé Jimma en Éthiopie.

**Méthodes:**

Une étude transversale a été réalisée du 1er au 30 Avril 2010. Un total de 343 patients diabétiques a été sélectionné au moyen d'une méthode d’échantillonnage systématique. Les données ont été recueillies au moyen de questionnaires structurés et d'un examen du carnet de santé ; les mesures anthropométriques ont été réalisées par des infirmiers formés.

**Résultats:**

L’étude a indiqué que 53% des sondés disposaient de connaissances relatives au diabète. L’étude a également montré que 64% des sondés étaient physiquement moins actifs, et que 17% des sondés marchaient moins de 30 minutes par jour. Seulement 18.1% des sondés étaient capables de maintenir leur glycémie à jeun à un niveau inférieur à 126 mg/dl.

**Conclusion:**

Cette étude illustre que le niveau de connaissances sur le diabète et les pratiques d'autosoins chez les patients diabétiques étaient faibles. Elle a également permis de montrer que le niveau d'activité physique, d’éducation des sondés et la dose d'agents hypoglycémiants oraux pris par les sondés affectaient de manière significative le contrôle glycémique.

## Introduction

### Background

Diabetes is becoming a pandemic disease resulting in an increased need for health care. Despite the great advancements that have been made in the treatment of diabetes in recent years, diabetes is one of the major causes of morbidity and mortality. It has a significant impact on the patients‘ quality of life, productivity and involves enormous health costs for virtually every society^1^. The situation in the developing world, particularly in Africa, is even worse caused by late diagnosis and poor access to diabetic care.^2^ In managing diabetic mellitus (DM) proper self-care practice and optimal glucose control is an essential cornerstone in achieving successful health outcomes.^1, 2^ DM is a life-long challenge that requires behavioural change and adequate self-care practices for better glycaemic control. In the absence of appropriate self-care practice, the desired therapy targets are difficult, or even impossible, to achieve. Glucose control is almost entirely in the hands of the patient who lives with this condition. The patient's motivation to eat, exercise, take medication, test glucose levels and maintain a healthy body weight all plays a significant role in the management of DM.^3–5^

The American Diabetes Association (ADA) publishes standards of medical care yearly to promote the importance of achieving optimal glycaemic control; this is achieved when glycosylated hemoglobin (HbA_1c_) is less than 7%.^4^ Comprehensive treatment includes lifestyle modifications, pharmacological control of hyperglycaemia, hypertension, hyperlipidemia and preventive care including monitoring for glycaemic control and adequate self-care.^4, 5^ Diabetes care is based on self-management by the patient and the quest for improving glycaemic control has made it clear that whatever the technical expertise applied, the outcome depends on willing cooperation by the patient. This, in turn, depends on an understanding of the risks of diabetes and the potential benefit of glycaemic control and other measures such as^6^:

- following a balanced diet and drug regimens
- examining one's own urine
- blood glucose monitoring
- administration of insulin
- maintaining a healthy weight
- going for regular health checkups
- early recognition of symptoms associated with glucose urea and hypoglycemia.

Self-care practices in diabetes are crucial to keep the illness under control and as much as 95% of the self-care is usually provided by the patients or their families. Self-care involves not only completing these activities but also considering the inter-relationships amongst them and implementing appropriate changes in the daily plan when necessary.^7, 8^ In order to perform effective self-care, the patient needs physical skills, cognitive function and an awareness of how psychological factors affect self-care. Maintenance of near-normal glycaemic control has been demonstrated to reduce the risk of diabetic associated vascular complications; self-care practice is crucial to maintain near-normal glycaemic control.^9^ Thus, the aim of the present study was to assess self-care practices and glycaemic control amongst adults with DM at a diabetic follow-up clinic at the Jimma University Specialized Hospital (JUSH) in southwest Ethiopia.

## Ethical considerations

The ethical issues of this study were reviewed and approved by the Ethical Committee of Jimma University. Verbal consent was sought from all the informed respondents before the start of each interview.

## Methods

### Setting

A facility-based cross sectional study was conducted amongst diabetic patients at the follow-up clinic of Jimma University Specialized Hospital in Jimma, Ethiopia, to assess the self-care practices and glycaemic control of patients. The data were collected over a period of 30 days from 01 April 2010 to 30 April 2010. Most of the patients were referred to a follow-up visit at JUSH. In addition, patients who are treated in the medical ward of the hospital as diabetic patient were also transferred to the diabetic clinic for follow-up care. Those diabetic patients were used to collecting their medication on a monthly basis. However, for some patients the frequency of their visits can vary depending on their blood glucose level. During the study period, 1816 diabetic patients were registered for follow-up care. There is no routine diabetic health education programme at the clinic but it is occasionally given by nurses who are not dieticians. The follow-up care is mostly provided by nurses with minimal support from physicians.

### Sample size

The sample size was calculated assuming a 50% proportion (*p*) of diabetic self-care practice, a 5% marginal error (*d*) and a confidence interval (CI) of 95%. Based on this assumption the sample size was calculated by a single population proportion formula: *n* = (Z_1-a/2_)^2^ *p*(1–*p*)/*d* ^2^. This yields a sample size of 384. Since the source population consisted of less than 10 000 respondents, the sample size was adjusted with correction formula *n*_f_ = *n*_i_/1 + *n*_i_/*N*, where *n*_f_ = the final sample size, *n*_i_ = initial sample size 384 and *N* = total diabetic patients (1613). Considering a 10% non-response rate, 343 diabetic patients were planned to be included in the study.

### Sampling procedures

A patient was included in the study if he or she was 15 years of age or older and has been part of a follow-up programme for at least 1 year at a diabetic clinic. A 1-year period was considered because patients who have adequate blood glucose level control were appointed to a maximum of 4 months so as to get their three successive Fasting Blood Sugar (FBS) or Random Blood Sugar (RBS) results to assess their glycaemic control patterns. Patients with mental health problems, hearing impairments or any other serious health problems and those patients who were unable to provide the appropriate information were excluded. A systematic sampling technique was used to select patients. The diabetic clinics provide their services only two days per week (i.e. Mondays and Tuesdays); on average 160 patients are treated per week whilst 640 patients are treated per month. Based on the decision to collect data over the course of one month, the sampling interval was determined by dividing the expected number of diabetic patients per month into the sample size (343) which gives a sampling interval of two. Thus, every other patient coming to the clinic for a follow-up service was interviewed until the total sample size was reached.

### Procedure

Patients were interviewed using structured questionnaires. Questionnaires were prepared in English and translated into Amharic and Afan Oromo (local languages) and translated back into English to check its consistency. The Amharic and Afan Oromo versions were used for data collection after pretesting on 10% of the actual sample size in other similar settings. These instruments were adapted from similar studies.^8, 10^ To identify the patterns of glycaemic control, patients’ charts were reviewed, retrospectively; the last three successive FBS or RBS results were recorded from the patient's card. Anthropometric measurements were used to assess the body mass index (BMI) of the patients which is calculated by dividing the weight of the subject in kilograms (kg) by the height of the subject in meters squared. The data were collected by trained nurses.

### Analysing

The data were analyzed by Statistical Package for Social Science (SPSS) version 16.0. Descriptive statistics were used for most variables such as socio-demographic data; a Chi-square test was employed to determine the presence of the association between glycaemic control and self-care practices with socio-demographic characteristics. Variables that showed significant association on bivariate analyses were fitted into a multi-variable logistic regression model. All statistical tests were two-sided and statistical significance was set at a *p*-value < 0.05.

### Operational definition

#### Glycaemic control

The level of glycaemic control was indicated as ‘adequate glycaemic control’ when FBS results were less than 126 mg/dL (7 mm/L) (i.e. an average of three visits), or when RBS results were less than 200/dL; ‘inadequate glycaemic control’ takes place when a parameter is beyond the criteria of adequate glycaemic control.

#### Knowledge

Knowledge of patients’ relating to diabetes and self-care practice was assessed by making use of ‘yes/no’ questions. A correct answer was coded as ‘1’ and an incorrect answer as ‘0’; the score was then computed. Respondents were labelled as having knowledge of diabetes and self-care practices if he or she scored ≥ the mean value, and having poor knowledge if he or she scored less than the mean value.

#### Physical activity

The levels of physical activity of the patients were classified into three levels based on their physical activities:

1. Light activity: Patients are in a sitting position most of the time, less than half of the time they are standing or walking, they seldom carry heavy things, and travel by car or motorbike.
2. Moderate activity: Patients are sitting, standing and walking about half of their time. They spend some time carrying heavy things and use public transport during non-leisure hours.
3. Heavy activity: Patients spend almost none of their time sitting and almost all of their time standing or walking, most of the time carrying heavy things, and they use public transport, cycle or walk everywhere. Regular exercise
4. (i.e. 20 min – 30 min of aerobic exercise such as walking or swimming 3–4 days per week) was also considered.

## Results

### Socio-demographic characteristics of the participants

A total of 343 diabetic patients participated in the study giving a response rate of 100%. Because one of the questionnaires was incomplete, one case was excluded from the analysis. The socio-demographic characteristics of the participants are presented in Table 1. More than half of the participants were male (206, 60.2%). The mean age of the participants was 45.2 ± 15.9; nearly half of them were older than 44 years (183, 53.3%).

**TABLE 1:** Socio-demographic characteristics of the participants (Jimma University Specialized Hospital in Ethiopia, April 2010).

Characteristic type	Characteristic	*f*	%
Gender	Male	206	60.2
	Female	136	39.8
Age	15–29	60	17.5
	30–44	99	28.5
	45–64	136	39.8
	> 65	47	13.7
Marital Status	Married	257	75.1
	Single	68	19.9
	Others†	17	4.9
Religion	Muslim	175	51.2
	Orthodox	131	38.3
	Others‡	36	10.5
Ethnicity	Oromo	209	61.1
	Amhara	65	19.0
	Keffa	23	6.7
	Dawro	13	3.8
	Others	32	9.4
Education	Illiterate and non-formal education	103	30.1
	Grade 1–6	77	25.7
	Grade 7–12	54	22.5
	> Grade 12	54	15.8

*Source*: Survey, 2010

*f*, frequency.

† Divorced and/or widowed.

‡ Protestant and/or Catholic.

#### Pattern of diabetes

Type 2 DM constituted 211 participants (61.7%) and the remaining 131 participants (38.3%) were type 1. The mean duration of diabetes since diagnosis was 5.8 years making the average age at diagnosis of diabetes about 44.18 years. The BMI of the respondents ranged from 15.02 kg/m^2^ to 35.56 kg/m^2^ and the mean BMI was 23.4 kg/m^2^ ± 4.22 kg/m^2^; only 5.8% of the patients had a BMI > 30 kg/m^2^.

### Knowledge about diabetic mellitus

The mean score in terms of knowledge items was 8.12 ± 1.678 with 53% of the participants scoring above the mean; 47% of the participants were scored below the mean value. Participants were asked whether DM was a chronic disease, or a curable disease and whether it is possible to control it by interventions, such as a healthy diet, exercise, and administering insulin and hypoglycemic drugs. Accordingly, 286 respondents (83.6%) responded that it is chronic disease, 215 respondents (62.9%) said that DM is not curable and 333 respondents (97.4%) reported that it is possible to control diabetes. Furthermore, the majority (70%) of the respondents had knowledge of the signs and symptoms of hypoglycaemia and 68.4% knew what care should be taken in the event of hypoglycaemia. However, only 48% of the respondents had knowledge about appropriate precautions for the prevention of hypoglycaemia. Similarly, 73% of the respondents said that they were not fasting for fear of hypoglycaemia whilst 27% stated that they were indeed fasting.

### Dietary knowledge of the respondents

Concerning the knowledge of respondents relating to food items that have an increased glycaemic index (which means that the blood glucose level is raised) and need to be cut down by diabetic patients, the majority of the respondents answered that *injera* (i.e. a stable food diet in Ethiopia made of Teff cereal which is the tiniest kind of cereals) and *kocho* (i.e. a traditional staple food made of a false banana plant called enset or *Ensete Scitamineae*) have a low glycaemic index and could be eaten freely by diabetic patients; almost all patients reported that sugar and honey containes a high glycaemic index. Only 93 respondents (27.2%) stated that fibrous food (e.g. whole grain cereals) has a high glycaemic index and similarly 133 patients were unaware that rice has a high glycaemic index. However, 288 respondents (84.2%) knew which diet to take when their blood glucose level was below normal and 275 respondents (74.3%) knew which food options should be eaten by diabetic patients. Furthermore, 245 respondents (71.6%) knew what the results were of skipping meals after taking injections or oral hypoglycemic drugs.

#### Glycaemic control and medication uses

At the time of the study, 341 respondents (99.7%) were on pharmacologic therapy for diabetes and of those who were on medication, 204 respondents (59.6%) were taking insulin alone; a total of 131 type I diabetic patients and 63 (29.8%) type II patients were taking insulin. However, 85 (24.9%) respondents, 53 (15.5%) respondents and 10 (2.9%) respondents were taking one oral hypoglycemic agent, two hypoglycemic agents, and insulin and oral hypoglycemic agents, respectively.

FBS was done for 333 (97.4%) of the respondents whilst RBS was done for the remaining respondents. The mean FBS was 176mg/dL ± 57.4. Glycosylated hemoglobin (HbAlc) was done for none of the respondents.

#### Self-care practice

The self-care practices of the diabetic patients are displayed in Table 2. Only 190 patients (55.6%) and 188 patients (55.0%) had regular meals including lunch time. Of the patients who were using insulin, only 157 (45.9%) took meals 30 minutes after each insulin injection, and the remaining patients were used to injecting themselves before meals or took meals after one hour of taking an injection. With regard to physical activity, the study showed that only 69 respondents (20.2%) reported that they were taking necessary precautions when engaged in unusual exercise or ‘first-time’ exercise. Similarly, 166 respondents (48.5%) walked for 15 min – 30 min per day.

**TABLE 2:** Self-care practices in diabetic follow-up clinic of Jimma University Specialized Hospital in Ethiopia (April 2010).

Self-care practice	Response category	*f*	%
**Dietary self-care practice**			
Regular time for meals	Yes	190	55.6
Regular time for breakfast	Yes	186	54.4
Regular time for lunch	Yes	188	55.0
Regular time for snack	Yes	95	27.8
Insulin Injection with respect to meal	30 minutes after injection	157	76.9
	After one hour	25	12.3
	Before injection	10	4.9
	At any time	16	5.9
**Physical activity self-care**			
Taking precaution when you are engaged into unusual exercise	Yes	69	20.2
Practice of waking on foot daily 3–4 days a week for 15–30 minutes	Yes	166	48.5
Level of physical activity	Light activity	221	64.6
	Moderate activity	95	27.8
	Heavy activity	26	7.6
Practice of regular exercise	Yes	222	64.9
Practice of daily foot care	Yes	284	83.0
Have you sustained a foot injury?	Yes	59	17.3
Action taken in sustained injury	Getting health care	24	42.0
	By traditional means	13	21.0
	Heals spontaneously	20	35.0
**Blood glucose monitoring self-care practices**			
Do you practice self-monitoring of your blood glucose levels?	Yes	9	2.6
Do you undergo blood glucose determinations during each visit?	Yes	338	98.8
How do you describe your adherence status to your medication?	As prescribed	319	93.3
	Only when I have poor symptoms	20	5.3
	It is adjusted based on my blood glucose	2	0.6
	Haphazardly	1	0.3
**Self-Care on SMBG**	Yes	10	2.9

*Source*: Survey, 2010

*f*, frequency; SMBG, self-monitoring of blood glucose.

#### Predictors of glycaemic control

Multivariate logistic regression was used to identify factors associated with achieving adequate glycaemic control. Accordingly, patients’ educational status, levels of physical activity, taking a single dosage of an oral hypoglycaemic agent (OHGA) and walking by foot for 30 minutes per day were significant predictors of glycaemic control (*p* < 0.05). Furthermore, having knowledge on the processes of the disease, self-care practices, physical activity, educational level, walking on foot, taking a single dose of oral hypoglycemic (OHGA) drugs, and taking drugs as prescribed were significant (*p* < 0.05) predictors in biviarate analysis. However, no statistically significant associations were observed between marital status, religion, income, living conditions, types of diabetes, occupation, BMI, and the duration of diabetes. In bivariate analysis, patients who had knowledge of diabetes were two times more likely to control their blood glucose level than those who were not knowledgeable (Crude Odd Ratio = 1.77, 95%; CI = 1.05–3.11). This association did not exist after adjustment for other variables.

**TABLE 3:** Predictors of glycaemic control amongst diabetic patients treated at Jimma University Specialized Hospital in Ethiopia (April 2010).

Variables	Category	Odd Ratio			

		COR		AOR	
	
		95%	CI	95%	CI
Knowledge of diabetes mellitus	Poor knowledge	1	–	1	–
	Good knowledge	1.77	1.05–3.11*	1.50	0.23–2.87
Educational status	Illiterate	1	–	1	–
	Informal education	1.84	0.45–7.51	2.41	0.56–0.400
	1–6 grade	1.81	0.73–4.46	1.71	0.67–4.360
	8–12 grade	4.17	1.79–12.71*	3.50	1.44–10.52*
	>12 grade	4.17	1.62–5.94*	3.00	1.14–6.97*
Overall self-care	Poor self-care	1	–	1	–
	Good self-care	1.96	1.04–5.61*	1.25	0.62–2.52
Level of physical activity	Less activity	1	–	1	–
	More activity	2.28	1.31–4.00*	2.43	1.31–3.50*
Walking by foot for 30 minutes	No	1	–	1	–
	Yes	1.34	1.12–2.47*	2.21	1.25–4.21*
Single dose of OHGA	No	1	–	1	–
	Yes	2.85	1.09–7.44*	3.50	1.27–7.63*

COR, crude odd ratio; AOR, adjusted odd ratio; CI, confidence interval; OHGA, oral hypoglycaemic agent.

* Significant at *p <* 0.05.

## Discussion

The present study attempted to assess diabetes mellitus patients’ knowledge and self-care practices in terms of living with the disease. According to the findings of the study, respondents’ choice to incorporate physical activities (such as walking on foot for 30 minutes per day) was a strong predictor of glycaemic control. In addition, many patients did not know which precautions to take when doing exercise. This finding is consistent with previous studies.^8, 11^ This study revealed that most of the respondents were less active and this could be because of having inadequate knowledge in terms of the benefits of regular physical exercise and a fear of hypoglycaemia. In this study, dietary self-care was inadequate which was also supported by the available literature^12, 13^ on the subject. The majority of the respondents did not self-monitor their blood glucose levels; the majority of the respondents did not get advice on the Self-Monitoring of Blood Glucose (SMBG). Access to SMBG remains very low as it has been reported in a previous study^14^ although those who had access to it were not using it. In this study, 98.8% of the respondents received SMBG service during their last three visits. This indicates that SMBG service is readily available for patients at hospital level.

Discussing the adequacy of glycaemic control will be a handicap without mentioning glycosylated hemoglobin (HbAlc) determination; the ADA recommends that a patient should have glycosylated hemoglobin determination^4^ at least twice yearly. In addition, a study conducted in the United States of America (USA) showed that at least 77% of diabetic patients had at least one glycosylated hemoglobin determination in the two years preceding the study.^15^ However, in contrast to previous studies, none of the patients had glycosylated hemoglobin. The study also revealed that the mean FBS was 176.7 ± 57.4 mg/dL which is better than some of the previous reports,^14^ but it is far higher than ADA recommendations which specify that good metabolic control or HbAlc level should be around or below 7%.^4^ The majority of the patients (82.9%) had FBS above the target level of 126 mg/dL as compared with 79% of the patients who had FBS greater than 120 mg/dL in another study^14^. Similar finding were reported in a previous study.^10^ This indicates that most of the patients were not controlling their blood glucose level, despite most of them taking medication provided free of charge; almost all the patients took their drugs as prescribed by a physician.

Patients taking oral hypoglycaemic agents appeared to have better glycaemic control than those taking insulin or a combination of oral glycaemic agents. The mean FBS in this group was 168.6 mg/dL as compared to 174 mg/dL for those taking insulin and 192 mg/dL for the combination of oral hypoglycaemic agents. The possible explanation for this is that patients taking a single oral agent (61.5%) had DM for at most 5 years whilst about 60% of patients insulin and 56.6% of patients taking combination of oral agents had DM longer than 5 years which means that good control in a single oral agent was due to the early disease than the effect of the treatment given. Patients taking a lower dose of oral agents had a far better FBS level than those taking higher doses and it is consistent with previous studies.^1^

Self-care activity in diabetic management includes medication self-care, dietary self-care, physical activity self-care and self-monitoring of blood glucose levels. The study showed that each self-care activity was not significantly predicting glycaemic control in the final regression model. But overall self-care activity was significantly associated with adequacy of glycaemic control. This finding was supported by another study^16^ wherein self-care involves not only completing each self-care activity but also considering the inter-relationship amongst them and implementing appropriate changes in patients’ daily plans.

## Conclusion

In general, self-care practice was inadequate, especially in terms of physical self-care activity and a deficit in terms of knowledge related to diabetes; this could be explained by factors such as limited education and low levels of educational status. Thus, health care providers should consider developing educational programmes and activities to educate patients on the prevention and treatment of diabetes, and should not rely on medical intervention only.

### Limitations of the study

The finding of this study may be interpreted with caution as it is facility-based which may produce more positive results. In addition, selection bias might be introduced as we have used systematic sampling on a small sample size. Self-reported data may also suffer from social desirable bias.
